# The diagnostic and prognostic role of miR-146b-5p in differentiated thyroid carcinomas

**DOI:** 10.3389/fendo.2024.1390743

**Published:** 2024-07-05

**Authors:** Carolina Ferraz, Gustavo Bittar Cunha, Mariana Mazeu Barbosa de Oliveira, Lucas Ribeiro Tenório, Adriano Namo Cury, Rosália do Prado Padovani, Laura Sterian Ward

**Affiliations:** ^1^ Thyroid Diseases Unit - Division of Endocrinology, Department of Medicine, Faculty of Medical Sciences/Irmandade da Santa Casa de Misericórdia de São Paulo, São Paulo, Brazil; ^2^ Division of Head and Neck Surgery, Department of Surgery, Irmandade da Santa Casa de Misericórdia de São Paulo, São Paulo, Brazil; ^3^ Laboratory of Cancer Molecular Genetics, School of Medical Sciences, State University of Campinas (UNICAMP), Campinas, São Paulo, Brazil

**Keywords:** thyroid cancer, diagnostic, prognostic factors, microRNA, MiR-146b

## Abstract

**Introduction:**

Samples classified as indeterminate correspond to 10-20% of cytologies obtained by fine needle biopsy of thyroid nodules, preventing an adequate distinction between benign and malignant lesions and leading to diagnostic thyroidectomies that often prove unnecessary, as most cases are benign. Furthermore, although the vast majority of patients with differentiated thyroid cancer (DTC) have such a good prognosis that active surveillance is permitted as an initial therapeutic option, relapses are not rare, and a non-negligible number of patients experience poor outcomes. MicroRNAs (miR) emerge as potential biomarkers capable of helping to define more precise management of patients in all these situations.

**Methods:**

Aiming to investigate the clinical utility of miR-146b-5p in the diagnostic of thyroid nodules and evaluating its prognostic potential in a realworld setting, we studied 89 thyroid nodule samples, correlating miR-146b-5p expression with clinical tools such as the 8th edition from the American Joint Committee on Cancer (AJCC/UICC) and the American Thyroid Association Guideline Stratification Systems for the rate of recurrence (RR).

**Results:**

miR-146b-5p expression levels distinguished benign from malignant thyroid FNA samples (p< 0.0001). For indeterminate nodules, overexpression of miR-146b-5p with a cut-off of 0.497 was able to diagnose malignancy with a 90% accuracy; specificity=87.5%; sensitivity=100%. An increased expression of miR-146b-5p was associated with greater RR (p=0.015). A cut-off of 2.21 identified cases with more vascular involvement (p=0.013) and a cut-off of 2.420 was associated with a more advanced TNM stage (p-value=0.047).

**Discussion:**

We demonstrated that miR-146b5p expression in FNA samples is able to differentiate benign from malignant indeterminate nodules and is associated with an increased risk of recurrence and mortality, suggesting that this single miRNA may be a useful diagnostic and prognostic marker in the personalized management of DTC patients.

## Introduction

A recent systematic review and meta-analysis shows that, regardless of the country’s development and economic situation, thyroid nodules are diagnosed in almost 25% of the world’s population and their incidence has been increasing in recent years (1). Differentiated thyroid carcinomas (DTCs) have a prevalence of approximately 5%, which makes them the most common malignant tumors of the endocrine system and one of the 10^th^ most common cancers in the population (2). According to the most recent consensuses, ultrasound (US) guided fine needle aspiration (FNA) is the procedure of choice for further evaluation of suspicious thyroid nodules (3, 4). Under optimal conditions, the cytology thus obtained classifies 60–80% of nodules as benign and 3.5–5% as malignant (5, 6). Unfortunately, up to 30% of the FNA samples are classified as indeterminate and cannot distinguish between benign and malignant lesions (5, 6). Due to the variety of diagnostic possibilities associated with indeterminate cytologies, patients with this cytological diagnosis are often subjected to diagnostic surgeries because of the risk of malignancy, which is benign in the vast majority of cases (5, 6). However, recent advances in available molecular tests have proven effective in further risk stratifying aspirates to improve the diagnostic certainty of indeterminate thyroid nodules and even have the potential to select patients for targeted therapy, leading to their recommendation for use in certain cases (3, 4).

On the other hand, although DTCs are mostly indolent and have excellent survival rates, up to 30% will have locoregional or distant metastatic recurrence 10 or 20 years after diagnosis (7). Furthermore, there is evidence of an increase in the incidence of advanced-stage disease in recent years, which confers a poorer prognosis (8). Molecular markers aimed at identifying these patients at early stages, thus optimizing the initial approach and perhaps reducing the risk of disease recurrence are extremely necessary.

After thyroidectomy, patients with DTC are currently assessed for mortality risk using the tumor-node-metastasis (TNM) classification according to the 8th edition of the American Joint Committee on Cancer (AJCC), which takes into account the patient’s age, size of the primary tumor, the number and location of metastatic lymph nodes, and the presence of distant metastases (3, 9). On the other hand, the American Thyroid Association (ATA) focuses on the risk of recurrence or persistence of the disease, proposing another stratification that divides patients into low, intermediate and high risk (3). This classification is based on histological characteristics such as size of the primary tumor, vascular involvement, extrathyroidal spread of the tumor, histological subtype, multifocal disease and lymph node involvement (3). The presence of BRAFV600E and TERT mutation is also validated as an additional tool in the prognosis of these patients, upstaging a low risk intrathyroidal <4 cm wild type BRAF papillary thyroid carcinoma (PTC) into high risk when these mutations are present (3). A third strategy is routinely employed in the follow-up of patients according to ATA guidelines: dynamic risk stratification of response to initial therapy (RCIT) (3). Periodic evaluation for biochemical or structural evidence of disease involves neck US, basal and stimulated serum thyroglobulin (Tg), as well as measurement of anti-thyroglobulin (anti-Tg) antibodies (3). Unfortunately, these tests have low positive predictive value and low specificity (10–12).

MicroRNAs (miRNAs) are small, non-coding RNAs that regulate protein expression and play important roles in many biological processes including oncogenesis, progression, angiogenesis and metastasis (13). miRNAs dysregulation has been actively investigated in search of biomarkers for early diagnosis, prognosis, monitoring and treatment of multiple types of cancer, including differentiated thyroid carcinomas (14). An increase in aberrant miRNA expression, particularly of miR-222, miR-221 and miR-146b-5p. has been demonstrated in PTCs compared to normal thyroid tissues, but their clinical utility remains to be further demonstrated (15–17).

To better describe the role of these miRNAs in clinical practice, our group has been studying the role of each of these miRNAs in a cohort of patients (data not yet published). Because miR-146b-5p appears very promising, we aimed to evaluate the performance of this unique miR-146b-5p expression in fine-needle aspiration biopsy (FNAB) material as a diagnostic and prognostic marker in patients with thyroid nodules in a real-world setting, in comparison to the two systems of currently used prognostic staging: TNM mortality risk (MR) and ATA risk of disease persistence/recurrence (RR).

## Materials and methods

### FNAB sampling and data collection

This study was approved by the local Human Research Ethics Committee of the Hospital Santa Casa de São Paulo (CAAE: 38456414.7.0000.5479), and written informed consent was obtained from all patients whose clinical samples were used in this research.

A total of 102 FNA samples were obtained from patients consecutively referred for thyroid thyroidectomy for nodular disease in Santa Casa de São Paulo, a tertiary care academic medical center. MR was assessed according to the 8th edition of the AJCC/UICC TNM classification, while RR was classified according to the last edition of the American Thyroid Association Guidelines3.

FNA nodule biopsy was performed intraoperatively with a 24-gauge needle inserted into the thyroid nodule under surgeon guidance, and two to three passes were used to collect the cytological specimen, which was suspended in TRIzol (Invitrogen), frozen, and stored at -80°C. Some nodules were diagnosed only intraoperatively and not seen before with ultrasound; thus, information about Bethesda category for these nodules was not available.

Another 40 patients also referred for surgery and whose final histological diagnosis was benign constituted the control group. Referral for surgery was based on assessment of the clinical characteristics of the nodule and personal preferences of each patient.

### RNA extraction, reverse transcription, and real-time PCR

FNAB samples in Trizol frozen at -80°C were used for total RNA extraction. Total RNA was extracted and reverse transcribed to cDNA according to standard protocol by the miRNeasy Micro Kit (Qiagen^®^) and the miScript^®^ II RT Kit (Qiagen^®^) respectively. Quantity and quality of RNA yield was assessed using the NanoDrop 2.000 (Thermo Scientific^®^). Reverse transcription was carried out for each miRNA using 7500 Real Time PCR System (Applied Biosystems) using a maximum of 200 ng of total RNA in 10uL for a 15µL final reaction of cDNA.

miRNA levels were determined in FNA specimens using RT-PCR by miScript^®^ SYBR^®^ Green PCR Kit (Qiagen^®^) according to manufacturer instructions. RT-PCR was carried out in an ABI PRISM 7500 Sequence Detection System. A total of 40 cycles of amplification were carried out, each cycle consisting of 15 seconds at 94°C, 30 seconds at 55°C, and 30 seconds at 70°C according to the standard protocol. SNORD95 was used as a normalizing gene, and Ct values were used for data analysis. All samples were evaluated in duplicated analysis.

### Statistical analysis

Statistical analysis of RT-PCR was achieved using 2^(-ΔCT)^ with a reference gene, and Mann-Whitney test to determine the significance of different levels of miRNA expression. We employed Receiver Operating Characteristic (ROC) curves to analyze the diagnostic efficacy of the differentially expressed miRNAs. ROC curves were constructed using XLSTAT software. The XLSTAT program utilizes the Youden Index to select the optimal cutoff point. Mann-Whitney tests was used to determine the significance of different levels of miRNAs expression.

## Results

The 89 evaluated cases included 49 patients whose characteristics are described in **Table 1** and who presented a profile compatible with the reality of cases currently observed in a tertiary medical center. However, after excluding samples with Ct values > 35 cycles in real-time PCR (RT-PCR), only 89 patients whose demographic and pathological characteristics are summarized in **Table 1** were further investigated.

**Table 1:** Demographic data of the thyroid nodules patients submitted to surgery and pathological features and demographic data of the malignant samples.

		Number of cases	(%)
Histology	Benign	40	44,94
	Malignant	49	55,06
Age	< 55 years	53	59,55
	> 55 years	36	40,45
Gender	Female	72	80,90
	Male	17	19,10
Histology	classic subtype -PTC	34	38,20
	follicular cell-derived neoplasms*	8	8,99
	FTC	1	1,12
	Insular subtype -PTC	1	1,12
	diffuse scleroing subtype PTC	1	1,12
	oncocytic subtype -PTC	3	3,37
	Warthin-smile subtype -PTC	1	1,12
	chronic thyroiditis	3	3,37
	Thyroid follicular nodular disease	27	31,46
	Oncocytic cell adenomas	1	1,12
	lipoadenoma	2	2,25
	follicular adenoma	7	7,87
FNA	Bethesda I	2	2,25
	Bethesda II	18	20,22
	Bethesda III	13	14,61
	Bethesda IV	7	7,87
	Bethesda V	18	20,22
	Bethesda VI	17	19,10
	Unclassified**	14	15,73
extra thyroid extension	Absent	32	65,31
	Present	17	34,69
vascular involvement	Absent	32	65,31
	Present	17	34,69
capsular involvement	Absent	31	63,27
	Present	18	36,73
multifocal disease	Absent	21	42,86
	Present	28	57,14
lymph node disease	Absent	34	69,39
	Present	15	30,61

*Include 5 non-invasive follicular thyroid neoplasm with papillary-like nuclear features (NIFTP) and 3 invasive encapsulated follicular subtype PTC.

**nodules incidentally diagnosed intraoperatively without previous cytological diagnosis.

Most of the patients were classified in TNM stage I, as shown in **Table 2**, had a low RR and presented an excellent evolution. Noteworthy, there were 38 (42.69%) FNA samples that were classified as Bethesda III, IV and V. Considering that the patients with Bethesda V would have been referred to surgery due to their high risk of malignancy, we further focused in the 20 patients (22.47%) whose cytology was considered indeterminate (Bethesda III and IV).

**Table 2:** Prognostic staging of the 49 thyroid cancer patients for their risk of mortality according to the 8th edition of the AJCC/UICC (TNM); for their risk of disease recurrence (RR) according to the American Thyroid Association Guidelines.

	Classification	Frequency	(%)
Risk of mortality (TNM)	I	41	83,67
	II	7	14,28
	IVB	1	2
Risk of recurrence (RR)	Low	23	46,94
	Intermediate	16	32,65
	High	10	20,41

**Figure 1:**
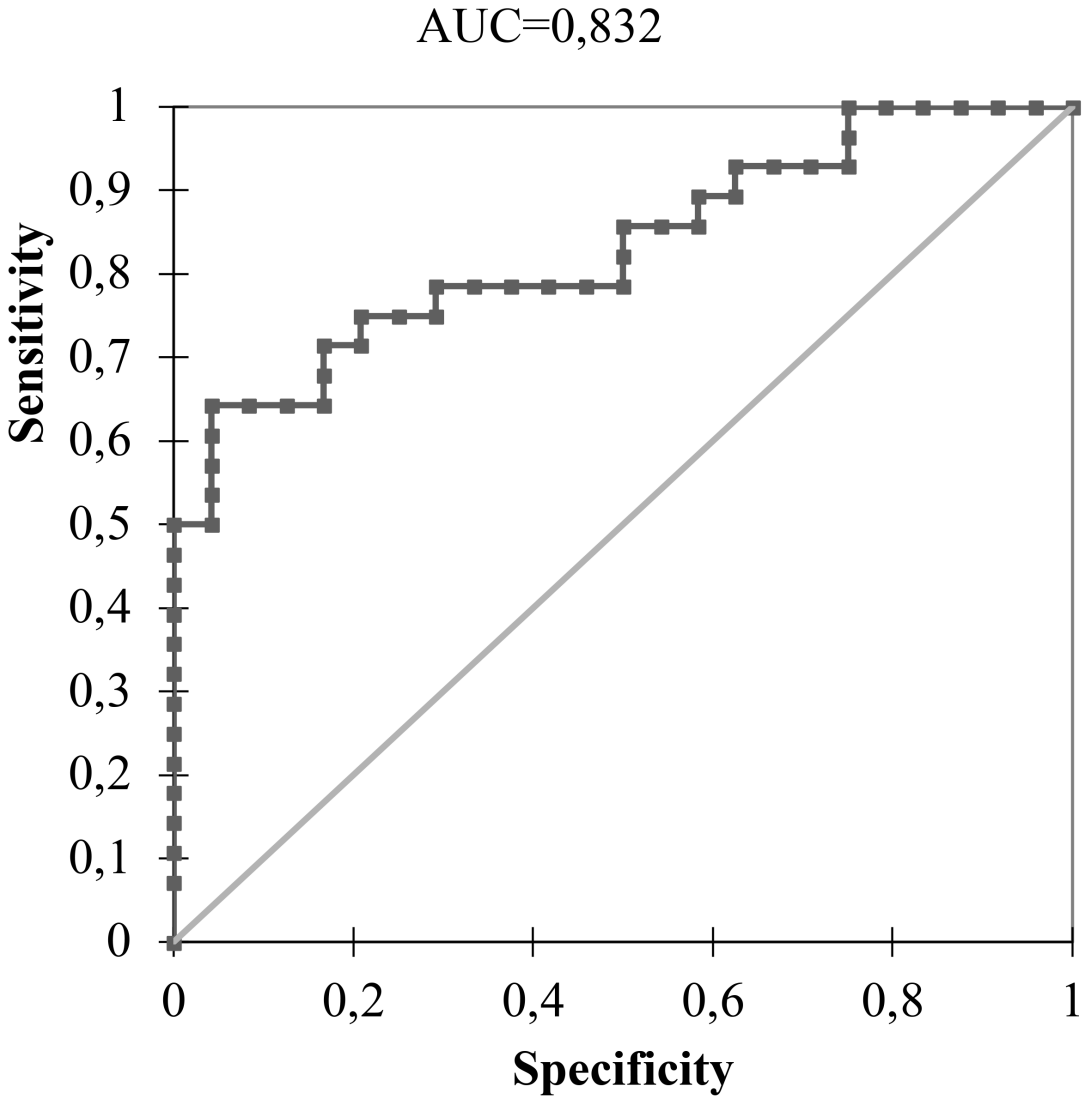
ROC curve of miR-146b-5p expression analysis in 89 thyroid nodules using a cut-off value of 0.497.

**Figure 2:**
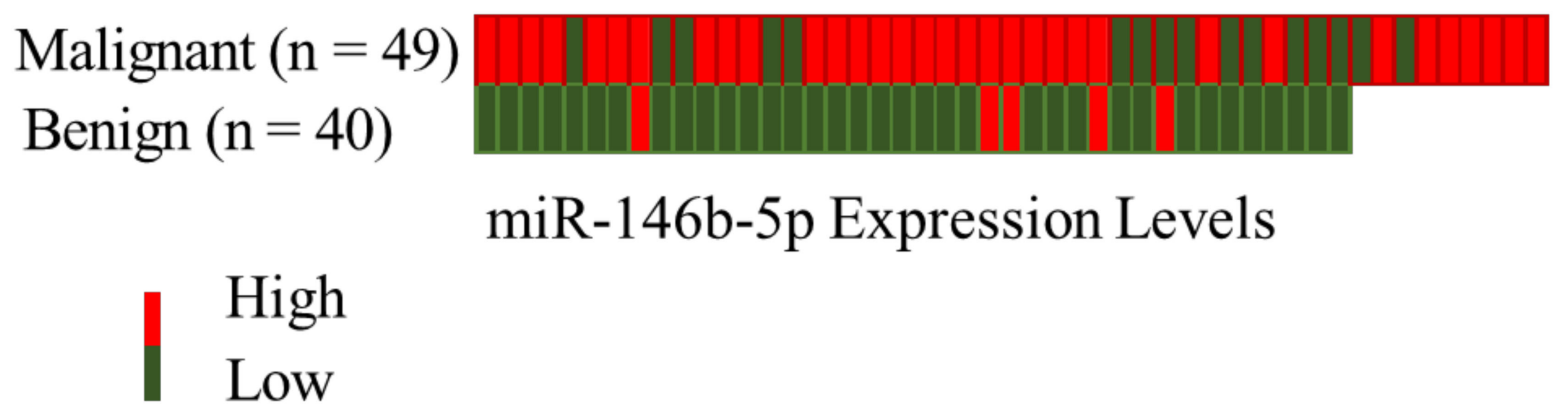
Heatmap representing the diagnostic profiles of benign and malignant 89 FNA samples according to miR-146b-5p expression considering a cut-off value of 0.497.

**Figure 3:**
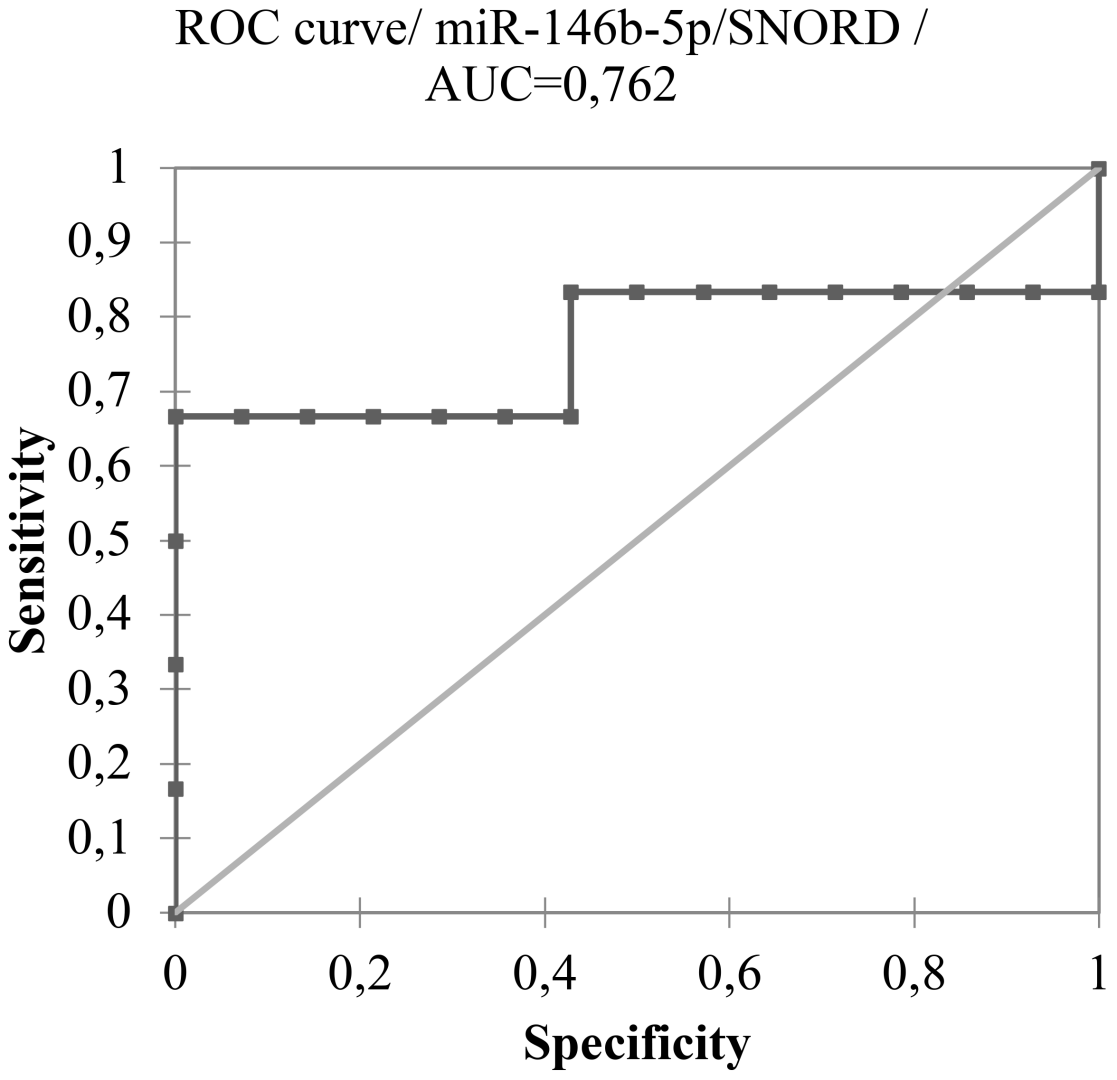
ROC curve of miR-146b-5p expression analysis of 20 indeterminate thyroid nodules FNA samples (Bethesda III and IV) considering a cut-off value of 0.497.

Using a cut-off value of 0.497, the expression of miR-146b-5p was able to differentiate malignant from benign tissues (p < 0.0001) with a specificity of 95,8% and sensitivity of 64,3% as shown in **Figures 1**, **2**. Concerning the 20 FNA obtained samples considered indeterminate, miR-146b-5p diagnosed all 4 malignant cases, with false positive results in only two of the 16 benign samples (accuracy = 90%; specificity = 87,5%; sensitivity = 100%) as illustrated in **Figure 3**. These two samples turned out to be a colloid nodule and chronic thyroiditis on histology.

**Figure 4:**
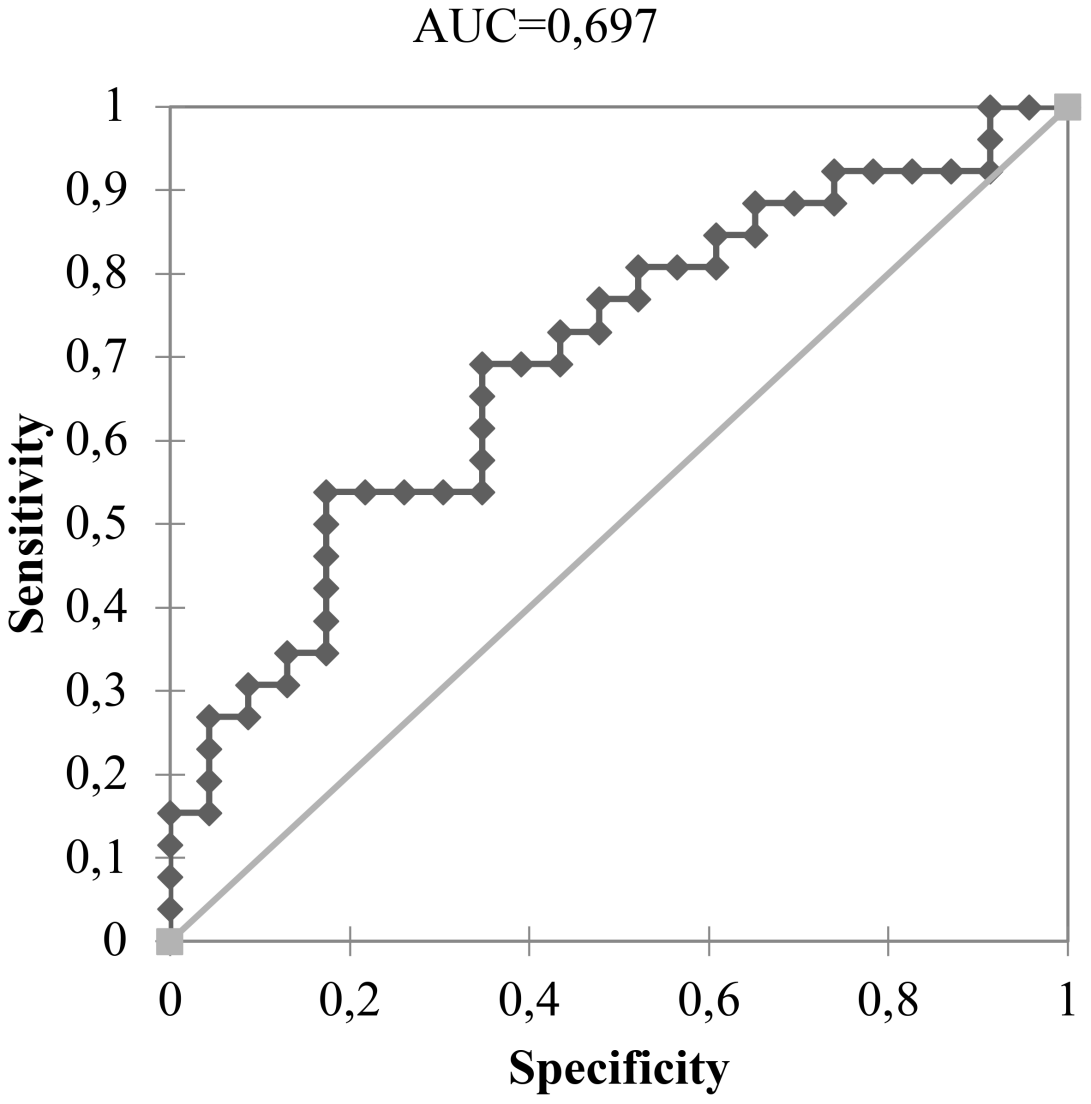
Expression values with 2(-ΔCT) > 2.21 of miR-146b-5p expression ability in the identification of 26 DTC patients with intermediate and high risk for recurrence.

Furthermore, an increased expression of miR-146b-5p was associated with higher RR (p = 0,015) as shown in **Figure 4**. In this context, miR-146b-5p expression values with 2^(-ΔCT)^ of 2.21 identified patients at intermediate and high risk for recurrence of DTCs with 81,8% specificity and accuracy of 64,4%. An analysis of histological variables showed that overexpression of miR-146b-5p (2(-ΔCT) > 2.21) was associated with vascular invasion (p=0.013). There was no significant association with the presence of extra thyroid invasion, capsular invasion, and lymph node metastasis or multifocal involvement.

**Figure 5:**
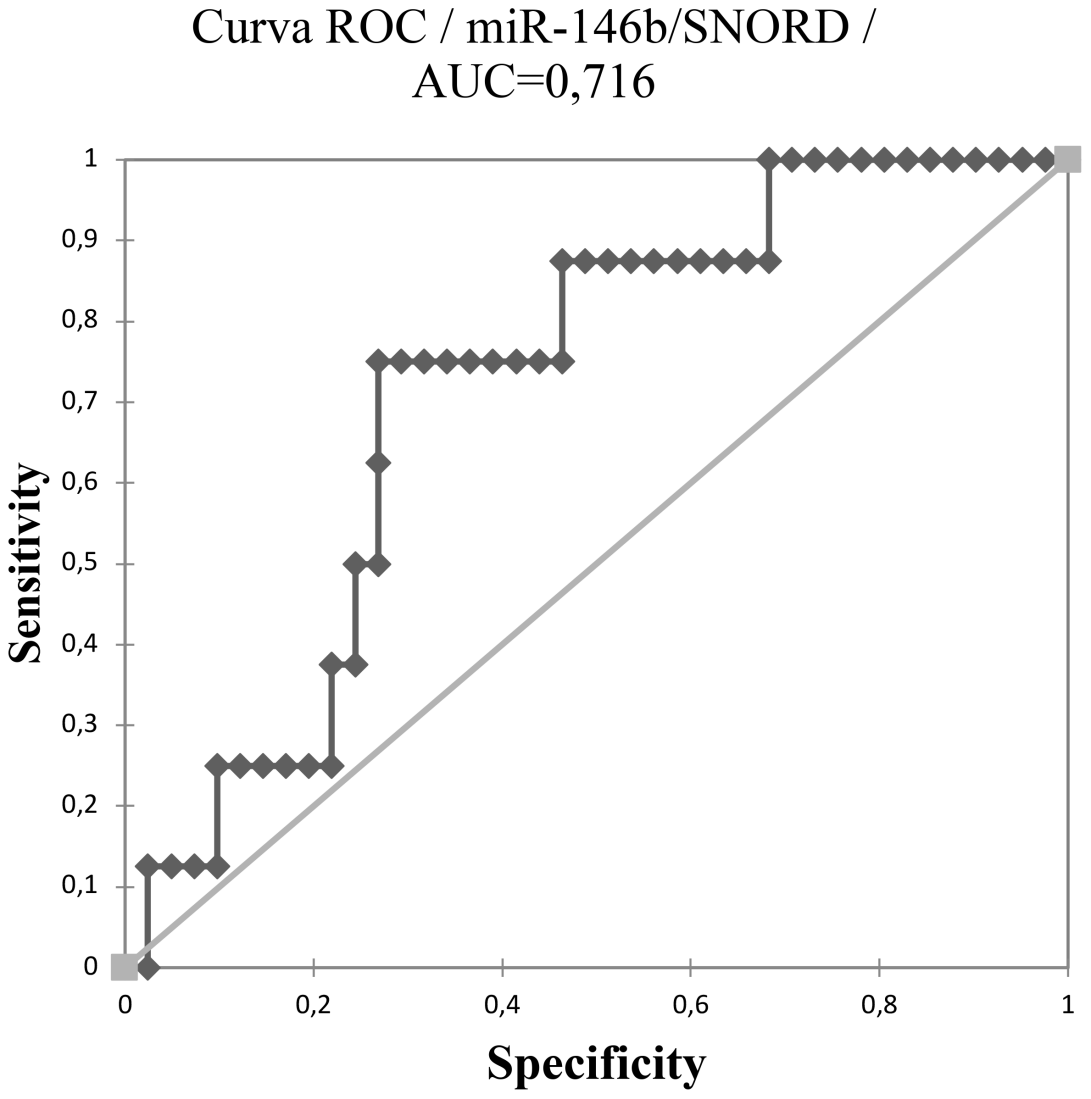
Expression levels of mir-146b-5p ability to identify the patient with worse TNM using a cut-off of 2.420.

Regarding the risk of mortality, an expression level of mir-146b-5p higher than 2.420 was associated with a worse TNM stage (p-value = 0.047) as shown in **Figure 5**.

## Discussion

The management of thyroid cancer is a good example of how “one size fits all” approaches have become personalized and precision medicine in our days. Molecular markers play a significant role in this context, both from a diagnostic and prognostic point of view. In this study, we demonstrated the clinical utility of mir-146b-5p as a diagnostic and prognostic marker in indeterminate thyroid nodules and in patients with DTC, respectively. Our findings demonstrate in our settings that a cutoff point > 0.497 can differentiate malignant from benign samples, and a cutoff point > 2.21 could modify the RR classification of these tumors.

According to Vargas-Salas et al, the ideal molecular test to evaluate indeterminate thyroid nodules should have a sensitivity above 90%, and specificity higher than 80% (18). Overexpression of miR-146b-5p proved to be a good diagnostic marker with accuracy = 90%; specificity = 87.5%; sensitivity = 100%.

The diagnostic role of miR-146b-5p for DTC have been endorsed by other groups. In 2017, Qiu et al. showed that the expression levels of miR-146b were significantly higher in thyroid carcinomas than in cancer-free tissues (19) . More recently, evaluating indeterminate thyroid nodules, Sponziello et al. have also found a high specificity (93%) and PPV (93%), for the overexpression of miR-146b-5p in malignant cases (20). A significant miR-146b-5p overexpression (> 30-fold) was also observed in PTCs compared to benign thyroid nodules by Agretti et al. (21). In agreement with our and other studies in FNA samples, Mahmoudian-Sani et al. showed that the up-regulation of miR-146b-5p (cut off = 1.02) was able to sort out malignant cases efficiently (p value = 0.001) (22).

Currently, two commercially available tests are globally recognized for utilizing a panel of miRNAs in indeterminate samples for diagnostic purposes: ThyraMir (23) and Mir-THYpe (24). Both assays incorporate the analysis of miR-146b-5p within the panels. ThyraMir assesses the expression of 10 miRNAs and, in its initial publication, exhibited a sensitivity of 89%, negative predictive value (NPV) of 94%, specificity of 85%, and positive predictive value (PPV) of 74% in indeterminate samples. Conversely, Mir-THYpe evaluates the expression of 11 miRNAs, with validation data correctly classifying 82 of 95 samples (23), yielding a sensitivity of 94.6%, specificity of 81%, NPV of 95.9%, and PPV of 76.1% (24).

We also demonstrated that higher levels of miR-146b-5p expression (cutoff > 2.21) were associated with more aggressive cases presenting vascular invasion, allowing a reclassification of the patient’s RR and TNM. Chou et al. found an association between the overexpression of miR-146b-5p in PTC patients with BRAF^V600E^ mutation and extrathyroidal invasion (25). In addition, Gómez et al. showed that miR-146b-5p expression had a positive correlation with thyroglobulin levels, even suggesting its possible utility in DTC patients’ follow up (17). Higher miR-146b-5p expression has also been associated to lymph node metastasis in PTC patients (16, 26). Furthermore, there is evidence that the overexpression of miR-146b-5p could decrease the radioiodine-sensitivity by regulation of the sodium-iodide-symporter (NIS) expression, which could influence therapeutic decisions involving radioiodine uptake (RAIU) (27). All these data, added to others that indicate that the overexpression of miR-146b-5pis harmful (17, 19, 28–30), reinforce our results that demonstrate that these patients are at greater risk of disease recurrence and a consequent worse outcome.

We aimed to reinforce the diagnostic and prognostic roles of miR-146b. However, it is also important to mention other potential roles identified in previous studies. miRNAs are known to regulate signaling pathways, such as MAPK, PI3K, AKT, GSK-3β/β-catenin, Wnt, mTOR, and NF-κB (31). Recent studies have suggested a possible relationship between miR-146b, BRAF, and RAS mutations. The V600E mutation in BRAF, associated with the upregulation of miR-146b, has been documented (32–34), and this association typically leads to more aggressive tumor behavior (32, 34). Interestingly, the study by Pamedytyte et al. suggests that the miRNA expression profile differs in PTC prone to recurrence compared to PTC that does not recur after the initial surgery (34). This finding indicates that the frequency of the BRAFV600E mutation does not reflect the recurrence status of PTC, thereby demonstrating an additional role of miR-146b beyond the BRAFV600E mutation (34). Basolo et al. found that RAS mutations increase the expression of miRNAs with recognized oncogenic roles in benign thyroid tumors (35). Therefore, when evaluating miRNA expression profiles in thyroid tumors, RAS status should be considered as a confounding factor. Additionally, they demonstrated that certain miRNAs, including miR-146b-5p, can distinguish between benign and malignant follicular-patterned neoplasms, despite morphological similarities and the shared presence of RAS mutations (35).

Our study has some limitations. Only 20 samples from our series were classified as indeterminate, making it necessary to analyze a larger sample to better validate the diagnostic power of miR-146b in these cases. Another important point is that the cut-off points found in our study only apply to our cohort. Therefore, they must be validated in other independent groups for generalizability.

To our knowledge, the data presented in this study are the first description of the clinical potential of miR-146-5p as a diagnostic and prognostic marker in a real-world setting. In contrast to most studies, which have sought to analyze a panel of miRNAs, we demonstrated that a single miRNA is capable of assisting in the diagnosis of indeterminate nodules and distinguish patients at higher risk of poorer outcome.

The high costs of molecular tests have been considered the main barrier to their indication in countries with limited financial resources (32). We suggest that the evaluation of a single miRNA may be sufficient to reduce the number of thyroid nodules diagnostic surgeries and optimize the management of DTC patients who will be better stratified, thus personalizing their treatment while reducing the costs involved in it.

## Data availability statement

The raw data supporting the conclusions of this article will be made available by the authors, without undue reservation.

## Ethics statement

The studies involving humans were approved by Human Research Ethics Committee of Hospital Santa Casa de São Paulo (Process 2015/00218-1). The studies were conducted in accordance with the local legislation and institutional requirements. The participants provided their written informed consent to participate in this study.

## Author contributions

CF: Conceptualization, Data curation, Funding acquisition, Investigation, Methodology, Project administration, Supervision, Visualization, Writing – original draft, Writing – review & editing. GC: Conceptualization, Data curation, Formal analysis, Investigation, Methodology, Software, Validation, Visualization, Writing – original draft. Md: Conceptualization, Data curation, Formal analysis, Investigation, Validation, Visualization, Writing – review & editing. LT: Conceptualization, Data curation, Investigation, Validation, Visualization, Writing – review & editing. AC: Conceptualization, Funding acquisition, Investigation, Project administration, Supervision, Visualization, Writing – review & editing. RP: Formal analysis, Investigation, Methodology, Project administration, Supervision, Validation, Visualization, Writing – original draft, Writing – review & editing. LW: Conceptualization, Data curation, Formal analysis, Methodology, Project administration, Supervision, Validation, Visualization, Writing – original draft, Writing – review & editing.
